# Postpartum women’s perception of antenatal breastfeeding education: a descriptive survey

**DOI:** 10.1186/s13006-020-00328-2

**Published:** 2020-10-14

**Authors:** May Loong Tan, Siew Cheng Foong, Jacqueline J. Ho, Wai Cheng Foong, Rokiah Mohd, Zuhaida Harun

**Affiliations:** 1 Department of Paediatrics, RCSI & UCD Malaysia Campus (formerly Penang Medical College), 4 Jalan Sepoy Lines, 10450 George Town, Penang Malaysia; 2 Department of Public Health, Penang State Health Department, George Town, Penang Malaysia; 3 Nutrition & Community Health, Penang State Health Department, George Town, Penang Malaysia

**Keywords:** Antenatal education, Breastfeeding, Baby friendly hospital initiative

## Abstract

**Background:**

Antenatal breastfeeding education (ANBE) is provided to all pregnant women attending Ministry of Health (MOH) clinics and some private health facilities in Malaysia, in line with the WHO/UNICEF Baby-Friendly Hospital Initiative (BFHI). However, the 6 month exclusive breastfeeding prevalence remains relatively low in Malaysia, suggesting that there may be a gap between what is currently taught and what is received by the women.

**Objectives:**

To determine how women perceived their ANBE experience in the first 8 weeks postpartum including what was useful and what they would like to have been included, sources of ANBE and infant feeding practices at the time of survey.

**Methods:**

Women during their first 8 weeks postpartum who attended MOH clinics in Penang State, Malaysia were surveyed using a self-administered questionnaire in April and May 2015. Categorical responses were presented as numbers and proportions while free text responses were compiled verbatim and categorised into themes. The perceptions of primiparous and multiparous women were compared. Multivariate logistic regression adjusted to known confounders was used to determine if ANBE was associated with exclusive breastfeeding at the time of survey.

**Results:**

A total of 421 women completed the 15-item questionnaire (84% response rate) of which 282 were complete and available for analysis. Of these, 95% had received ANBE, majority (88%) from MOH clinics. Almost all women found it useful. However, there were areas both in the delivery (e.g. too short) and the content (e.g. nothing new) that were described as not useful; and areas they would like more coverage (e.g. milk expression, storage and overcoming low milk supply). The exclusive breastfeeding prevalence at the time of survey was 61%. ANBE was significantly associated with exclusive breastfeeding even after adjusting for confounders (adjusted odds ratio [aOR] 8.1, 95% confidence interval 1.7, 38.3).

**Conclusions:**

ANBE is widely implemented and perceived as useful and may be associated with exclusive breastfeeding. Our findings give insight into content that women would like more of and how delivery of ANBE could be improved, including individualized sessions and communicating at a suitable level and language. Future studies could focus on the quality of ANBE delivery.

## Background

Antenatal breastfeeding education (ANBE) equips the mother-to-be with information and skills necessary for breastfeeding. It is also an opportunity for healthcare professionals to engage with pregnant women and their families at a time when many decisions about infant feeding are being pondered on. While breastfeeding is a natural process, it is not as simple as putting the baby to the breast [1] and only about two thirds of women who wanted to breastfeed actually achieved their intended duration for exclusive breastfeeding [2]. The original World Health Organization/United Nations Children’s Fund (WHO/UNICEF) Baby-Friendly Hospital Initiative (BFHI) included antenatal breastfeeding education for all expectant women in its “10-steps to successful breastfeeding” [3]. Moderate quality evidence from systematic reviews found that ANBE increases breastfeeding initiation rates and reduces the use of formula milk, but it is not known whether it increases duration of breastfeeding [4–6]. The updated WHO guidelines on breastfeeding continues to recommend “where facilities provide antenatal care, pregnant women and their families should be counselled about the benefits and management of breastfeeding” [7].

Malaysia adopted the BFHI policy in 1993 [8]. Since then, all Ministry of Health (MOH) health facilities in the country provide antenatal breastfeeding education. ANBE is provided to all pregnant women registered with any MOH health facility. It is common practice for all pregnant women to have contact with a MOH health clinic in the community, also known as Health Clinics (Klinik Kesihatan) and Maternal and Child Health Clinics (Klinik Kesihatan Ibu & Anak), where she would receive her antenatal care and immediate postpartum care as well as immunisation for her child. Each woman who registers for antenatal care at these clinics would be given a home-based record book which records her pregnancy progress, antenatal vaccination and ANBE attendance record. ANBE at these facilities is delivered by nurses and conducted either on a one-on-one basis or in a small group based on the topics listed in the national guide (See Table 1). All nurses delivering ANBE would have undergone the WHO 20-h breastfeeding course and each clinic would be provided with the teaching notes and material. These nurses would deliver the ANBE contents over a period of time, starting at the first antenatal visit (usually before 12 weeks of pregnancy) and complete all the topics before 32 weeks gestation. Once a topic has been covered, the nurse would record it in the woman’s home-based record book. During the study period, the content of ANBE was based on the National Guide for Implementation of BFHI policy [9].

**Table 1:** List of antenatal breastfeeding education topics given at all MOH facilities^a^

Topic No.	Title
1	Importance of breastfeeding to the baby
2	Importance of breastfeeding to the mother
3	Importance of early skin-to-skin after delivery
4	Importance of early initiation of breastfeeding
5	Importance of 24 h rooming-in
6	Importance of breastfeeding on demand or baby-led feeding or when the baby shows feeding cues
7	Importance of breastfeeding frequently to ensure adequate milk supply
8	Importance of good positioning and attachment during breastfeeding
9	Importance of exclusive breastfeeding for the first six months with no other food or drink
10	Importance of breastfeeding beyond six months with complementary feeding
11^a^	How to ensure early breastfeeding can be started
12^a^	Risks of formula or replacement milk feeding
13^a^	Implementation of mother-friendly care

*Extracted from: Panduan Pelaksanaan Inisiatif Hospital Rakan Bayi Kriteria Baru WHO/UNICEF 2009. In: Ministry of Health M, editor. 2013*

^a^During our study period, teaching of Topics 11 to 13 had not been implemented yet

There are also private establishments such as private hospitals or breastfeeding interest groups that provide ANBE with variable content, not necessarily following the MOH or WHO module. Lactation consultants such as those certified by the International Board of Certified Lactation Consultant (IBCLC) are not widely available. Therefore, it would be very uncommon for a mother to be given breastfeeding education by a lactation consultant.

Despite the fact that ANBE is widely available, breastfeeding rates in Malaysia remain low, especially the rates for exclusive breastfeeding. In 2016, the National Health and Morbidity survey found that the exclusive breastfeeding prevalence of infants under 6 months, (based on the WHO recommendation for household surveys), was 47.1% [10, 11]. Although this prevalence was higher than the 40% global average at that time, it still fell short of the WHO Global Breastfeeding target 2025 of at least 50% [12].

From local studies, several factors such as ethnicity, employment and parity have been shown to influence exclusive breastfeeding [13] and one study has suggested that attending ANBE sessions does not increase exclusive breastfeeding [14]. However, attendance might not be what matters but rather, the information that is delivered, how it is delivered and how it is received by a woman. This in turn might be dependent on her prior understanding and whether the information met her needs. Therefore, we hypothesized that there may be a gap between what is currently taught in our ANBE and what is received by the women. We conducted this study to determine how women perceived their ANBE experience in the first 8 weeks postdelivery, specifically what content was useful to them and whether there was any content they felt should have been covered. In addition, we determined where these women sought additional breastfeeding information after discharge from the hospital, and feeding practices at the point of the study.

This study was registered with the National Registry of Medical Research and ethical approval was obtained from the Medical Research Ethics Committee, Ministry of Health, Malaysia (NMRR No: 14–380-20,399).

## Methods

This was a descriptive observational study using a questionnaire to collect data from a sample of women from all 35 MOH Klinik Kesihatan and Klinik Ibu & Anak in the state of Penang, Malaysia (hereinafter referred to as ‘MOH clinics’) conducted in April and May 2015.

Women with infants not older than 8 weeks, attending their first postnatal visit or infant’s first immunisation at these MOH clinics were eligible for the study. With this timing, women would be in a position to reflect on the value of the ANBE they had received now that they were breastfeeding and specifically what ANBE content had helped. There were no exclusion criteria. Women meeting the eligibility criteria were approached by the attending staff and given information about the study. Those who consented proceeded to complete a 15-item self-administered questionnaire which took approximately 15 min. The staff were instructed not to influence the women’s responses. The questionnaire was developed by the investigators using an iterative process, piloted on a small sample of women and modified according to feedback obtained. It was made available in the three major languages used in Malaysia (i.e. English, Malay and Chinese). The first section of the questionnaire consisted of background questions such as age of both mother and baby, mother’s ethnicity, education level, occupation and parity. The next section was on whether the woman had received any ANBE and at which health facility. If the woman had received ANBE from one of the MOH clinics, the home-based record book was reviewed for the record of topics covered (see Table 1 for list of topics). The next section was about their perceptions of the usefulness of the ANBE and their reasons behind them (selected from a given list). A free text question sought if there was anything else the woman would have liked to have been included (see Supplementary Table for the questions in this section). The last section of the questionnaire was about additional sources of breastfeeding information they had sought, current infant feeding practice and reasons if they were not exclusively breastfeeding.

Exclusive breastfeeding is defined as the infant receiving only breastmilk either directly from the breast or expressed with no other food and drink including water [11].

### Sample calculation

We aimed to have 377 respondents for this survey. In order to obtain a sample that would be representative of the target population (i.e. women who deliver in Penang) we based our calculation on the 95% confidence interval and 5% confidence level of the number of live births of 20, 596 over a one-year period in Penang using a survey sample size calculator [15, 16]. A total of 500 questionnaires were distributed to account for the possibility of approximately 30% un-usable questionnaires.

### Statistical analysis

We tabulated the responses and presented numbers and proportions. Free text responses of content the women would have liked to have been included in ANBE were compiled verbatim and categorised into themes. We compared the ANBE perceptions of primiparous and multiparous women using Chi square or Fisher Exact test as there is evidence that their breastfeeding outcomes are different [17]. Univariate logistic regression was used to determine if ANBE was associated with exclusive breastfeeding at the time of survey and presented as a crude odds ratio (cOR) with 95% confidence interval (CI). Multivariate logistic regression adjusting for known confounders (ethnicity, parity and employment) was used to determine if ANBE was associated with exclusive breastfeeding at the time of survey and presented as an adjusted odds ratio (aOR) with 95% CI. Missing data were excluded. All statistical analysis was done using Minitab 16 and Stata 13 [18, 19]. A *p*-value of < 0.05 was taken as the level of significance.

## Results

Of the 500 questionnaires distributed, a total of 421 questionnaires were retrieved (84% response rate) and 282 had usable data. We excluded a large number of questionnaires because the ages of the infants were more than 8 weeks and hence did not qualify for inclusion. The majority of women who responded were between 26 and 35 years old, multiparous, employed and had at least a secondary education. The mean age of the babies at the time of study was 37 days (SD 11.6). Further details of the women are shown in Table 2.

**Table 2:** Characteristics of women (*n* = 282)

Characteristics	*n* (%)
Woman’s age	
< 25 years	41 (15)
26–30	128 (45)
31–35	80 (28)
> 35	32 (11)
Infant’s age (mean, SD)	37 days, 11.6
Ethnic background	
Malay	179 (63)
Chinese	70 (25)
Indian	25 (9)
Others	7 (3)
Education level	
None	1 (0.4)
Primary	8 (3)
Secondary	57 (20)
Tertiary	214 (76)
Primipara	107 (38)
Working woman	162 (57)
Received ANBE	267 (95)

Ninety five percent of them (*n* = 267) reported that they had received antenatal breastfeeding education (ANBE). The vast majority received ANBE from only MOH clinics (88%) while a small proportion received ANBE from only private health facilities (5%) and from both (5%); with the remaining without an answer. Among those who received ANBE at MOH clinics, between 86 and 99% of women had been taught at least one of the topics in the MOH module and 80% of women were taught all of the topics. There were no differences between primiparous and multiparous women (Table 3).

**Table 3:** Proportion of women who received ANBE from MOH facilities that completed the MOH ANBE Module

Topics	All women who had ANBE from MOH facilities (*n* = 235) *n* (%)	Primipara (*n* = 90) *n* (%)	Multipara (*n* = 158) *n* (%)
Topic 1	243 (98)	89 (99)	154 (97)
Topic 2	237 (96)	88 (98)	149 (94)
Topic 3	234 (94)	85 (94)	148 (94)
Topic 4	233 (94)	85 (94)	148 (94)
Topic 5	228 (92)	84 (93)	143 (91)
Topic 6	229 (92)	85 (94)	144 (91)
Topic 7	229 (92)	82 (91)	147 (93)
Topic 8	229 (92)	83 (92)	146 (92)
Topic 9	232 (94)	84 (93)	148 (94)
Topic 10	217 (88)	77 (86)	140 (89)
Topics 1–10	198 (80)	70 (78)	127 (80)

### Women’s perception of ANBE

Of the 267 women who received ANBE, 99% of them reported that the information they received was useful and the remaining did not answer the question. The top three reasons for perceiving the ANBE to be useful were: being told of the benefits of breastfeeding; how to position the infant for breastfeeding; and how to recognise correct attachment. However, there were 47 responses to the question why they thought the ANBE was not useful, indicating that some women who had perceived ANBE to be useful also thought that there were aspects that were not useful. The main reasons given for this perception were: the information given during ANBE was nothing new; duration was too short; materials used were not effective; and ANBE delivered in language they could not fully understand. The perceptions of the ANBE did not differ between primiparous and multiparous women. There were 42 responses to the free text question on topics women wished were covered. The most commonly requested topics were on milk expression techniques and expressed breastmilk storage and overcoming low milk supply. There were more multiparous women who wanted these two topics but the difference was not statistically significant. See Table 4.

**Table 4:** Perceptions of ANBE

	**All** **(*n*** **= 282)** ***n*** **(%)**	**Primipara** **(*n*** **= 107)** ***n*** **(%)**	**Multipara** **(*n*** **= 175)** ***n*** **(%)**
Found ANBE useful because			
(a) I was told about the benefits of breastfeeding	196 (70)	79 (74)	117 (67)
(b) I was taught how to position my baby	123 (44)	49 (46)	74 (42)
(c) I was taught how to know if my baby was attached/latched correctly	110 (39)	45 (42)	65 (37)
(d) I was taught how to deal with breastfeeding problems	99 (35)	43 (40)	56 (32)
(e) I was told how frequent I should feed my baby	104 (36)	43 (40)	61 (34)
(f) I was taught how to express milk	91 (32)	36 (34)	55 (31)
Found ANBE not useful because			
(a) I did not understand what was taught because of language	5 (2)	2 (2)	3 (2)
(b) The topics were nothing new to me – I already know them all.	21 (7)	5 (5)	16 (9)
(c) It was too short	16 (6)	7 (7)	9 (5)
(d) The people who taught the subject didn’t know what they were doing	2 (1)	1 (1)	1 (1)
(e) The material used to teach was not effective	6 (2)	1 (1)	5 (3)
**Responses to the question “What did you wished was taught to you during ANBE?”**	**All** **(*n*** **= 42)** ***n*** **(%)**	**Primipara** **(*n*** **= 12)** ***n*** **(%)**	**Multipara** **(*n =*** **30)** ***n*** **(%)**
Milk expression techniques and expressed breastmilk storage	19 (45)	4 (33)	15 (50)
Overcoming low milk supply	10 (24)	2 (17)	8 (27)
More about benefits of breastfeeding	5 (12)	2 (17)	3 (10)
Management of common breastfeeding problems	3 (7)	1 (8)	2 (7)
Positioning of infant	2 (7)	1 (8)	1 (7)
Exercise	1 (2)	1 (8)	0 (0)
Baby care	1 (2)	1 (8)	0 (0)

### Infant feeding practices up till 8 weeks postdelivery

At the time of the survey, 61% of the women were still breastfeeding exclusively. We conducted univariate and multivariate logistic regression to explore if attendance and perception of ANBE were associated with exclusive breastfeeding at the time of survey. Having attended ANBE was associated with higher odds of exclusive breastfeeding at the time of survey (cOR 11.4, 95% CI 2.5, 51.5). After adjusting for ethnicity, parity and employment, the odds of exclusive breastfeeding with ANBE remained high (aOR 8.1, 95% CI 1.7, 38.3). Women who perceived ANBE to be useful because they were taught how to overcome breastfeeding problems and how often to feed baby also had higher odds of exclusive breastfeeding at time of survey (cOR 1.9, 95% CI 1.1, 3.2 and cOR 1.8, 95% CI 1.1, 3.1 respectively).

Of those who did not exclusively breastfeed (39%), either one or both of these reasons were most frequently given: insufficient milk (60%) and having to go back to work (46%).

### Additional sources of information for breastfeeding skills and knowledge

**Fig. 1:**
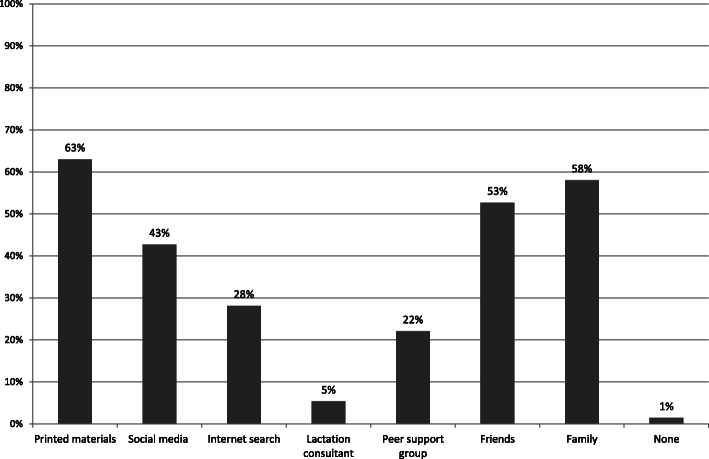
Other sources of information on breastfeeding (*n* = 281)

Almost all women reported use of other sources of information for increasing their breastfeeding skills and knowledge. Apart from the traditional sources of information such as printed materials and family, a large proportion used social media and the internet. Breastfeeding support groups were also relatively common and only a very small proportion sought the services of a lactation consultant. See Fig. 1.

## Discussion

Malaysia is one of the earliest countries to adopt and implement the BFHI [20]. Since then, the country has made considerable progress socioeconomically including better education and status of women [21]. With these improvements, it is likely that women have greater prior knowledge and their needs could have changed with time just as in other countries [22]. Although ANBE content has since been revised and updated following BFHI updates, it may be time to re-evaluate the method of ANBE delivery.

A systematic review and a large cluster-randomised trial showed that ANBE and the entire BFHI package increases initiation and duration of exclusive breastfeeding [6, 23].

While we found that there may be an association between ANBE and exclusive breastfeeding in our study population, the actual exclusive breastfeeding prevalence measured at up to 8 weeks in our study was only 61%. It is likely that this figure would drop further by the time the infant reaches 6 months old. Therefore, ANBE did not seem to have prevented the women in our study from prematurely stopping breastfeeding. However, we do not know if this was due to the quality of the ANBE delivery, its content or other factors.

There are a number of published studies evaluating mothers’ perception of antenatal education. Some of them evaluate the perception of the entire antenatal education package which would include education about breastfeeding [24, 25]. However, there is one study that evaluated perceptions after women had the opportunity to reflect on what the content meant to them now that they were breastfeeding. This study evaluated the perceptions of mothers who had used a web-based ANBE programme at 6 weeks postpartum and reported that the women found information about latching and positioning as well as benefits of breastfeeding as most helpful [26]. This was similar to the findings from our study.

A systematic review of qualitative studies on quality of antenatal education in general found that women prefer to have one-to-one interaction; opportunities to discuss and interact with other women; and having information given to her in a way that allows her to make her own decision rather than following a set of directives [27]. Although our study was not a qualitative study, the responses of some women gave an indication that the method of delivery should be reviewed. This could include checking for prior knowledge, allowing time for questions, avoiding overload of information at any one session and speaking at the level or language that the woman is able to understand.

Antenatal breastfeeding education in the MOH clinics was delivered in Malay language. While most women can speak some degree of Malay, those of Malay ethnic background whose mother tongue is Malay would better understand the content. Malay ethnicity has been found to be a significant factor associated with exclusive breastfeeding in Malaysia [13]. This certainly suggests that a woman’s preferred language needs to be considered when delivering antenatal breastfeeding education.

Our findings also suggest that the current ANBE module seemed to lack coverage on information regarding how to sustain breastfeeding and this is particularly important for a community with a high female workforce ratio such as Penang [28]. The current ANBE has a topic on the importance of breastfeeding beyond 6 months with complementary feeding (Topic 10) but it may not be detailed enough to prepare the woman to continue breastfeeding when she returns to work (typically at 60 days postpartum in Malaysia). Most of the information on milk expression and storage is given after delivery of the baby in current practice. Perhaps, this information could be better delivered as part of ANBE, taking into account whether or not the woman intends to return to work and reinforced before she leaves the hospital.

Other methods of delivering the content of ANBE should also be explored. For example, breastfeeding support groups could be recruited to complement the ANBE delivered within health facilities. Antenatal breastfeeding educators could also strive to increase their presence in social media (such as Facebook or Instagram) which is increasingly popular; or they could direct women to useful and reliable materials online. Social media is largely uncurated and users may not be able to discern reliable information. It is known that the infant formula industry uses social media to promote formula feeding and compliance to the infant formula market code of ethics is very low in this media [29]. When considering other options for delivering ANBE, perhaps a combination of online and face-to-face education may be better received by women here as there is evidence that even among researchers in Malaysia, they would rather ask people for answers to their queries than to find the answers themselves [30].

It is interesting to note that we found lactation consultant service was not a common source of information about breastfeeding. This is most likely because there are very few certified lactation consultants such as IBCLCs and there is little recognition for them in the Malaysian healthcare system.

The main limitation is that our questionnaire consisted mainly of closed ended questions. A qualitative study may give further insight. Recall bias might be perceived as a study limitation. However, in this case, what women were able to recall during the postpartum period could reflect what they valued from the ANBE they received and the quality of delivery.

## Conclusion

We found that ANBE was widely implemented, perceived as useful and having received it appears to be associated with exclusive breastfeeding up to the first 8 weeks postpartum. Our findings give insight into content that women would like more of and how delivery of ANBE could be improved such as individualized education and communicating at the level and language suitable to the woman. Apart from looking at other factors that affects exclusive breastfeeding rates, future studies could focus on the quality of antenatal breastfeeding education delivery.

## Supplementary information

**Additional file 1: Supplementary Table**.

## Data Availability

Not applicable.

## Abbreviations

ANBE: Antenatal breastfeeding education
MOH: Ministry of Health
BFHI: Baby friendly hospital initiative
WHO: World Health Organization
IBCLC: International Board Certified Lactation Consultant
