# Supplementary material for: Postpartum women’s perception of antenatal breastfeeding education: a descriptive survey
Source: Int Breastfeed J. 2020 Oct 14;15:85. doi: 10.1186/s13006-020-00328-2 (PMC7557059; doi:10.1186/s13006-020-00328-2)
Supplement: Supplementary file 1 — Additional file 1: Supplementary Table. [file 13006_2020_328_MOESM1_ESM.docx]

**Supplementary Table: Key questions on perception of ANBE**

| 1. Was the breastfeeding information you received during these teaching sessions (while you were pregnant) useful in helping you breastfeed? |
| --- |
| 1. If yes, what was most useful? (select one) |
| (a) I was told about the benefits of breastfeeding |
| (b) I was taught how to position my baby |
| (c) I was taught how to know if my baby was attached/latched correctly |
| (d) I was taught how to deal with breastfeeding problems |
| (e) I was told how frequent I should feed my baby |
| (f) I was taught how to express milk |
| (g) Others- specify |
| 1. If no, why? (select one) |
| (a) I did not understand what was taught because of language |
| (b) The topics were nothing new to me – I already know them all. |
| (c) It was too short |
| (d) The people who taught the subject didn’t know what they were doing |
| (e) The material used to teach was not effective |
| (f) Others – specify |
| 1. Was there anything else about breastfeeding you wished they had told you about during your pregnancy?   Free text answer |
